# Next Steps in Improving Telestroke Systems of Care—From Reach to Efficiency

**DOI:** 10.1001/jamanetworkopen.2025.34282

**Published:** 2025-09-02

**Authors:** Laura K. Stein, Kori S. Zachrison

**Affiliations:** Department of Neurology, Icahn School of Medicine at Mount Sinai, New York, New York (Stein); Department of Emergency Medicine, Mass General Brigham and Harvard Medical School, Boston, Massachusetts (Zachrison).

Telestroke is evidence based, cost-effective, and widespread in the modern stroke system of care.^1–3^ Patients presenting to rural and underresourced hospitals can access cerebrovascular expertise for decision-making related to time-sensitive acute reperfusion therapies in the absence of in-person consultants. However, robust assessment of telestroke network performance remains inherently challenging in the distributed hub-and-spoke model of care. Capturing telestroke use and meaningful process and outcome measures across systems of care can be especially difficult.^1^ We commend Stamm et al^4^ for successfully addressing this challenge. The authors performed an analysis of patients with acute ischemic stroke potentially eligible for thrombolysis treated with and without telestroke in the Paul Coverdell Michigan Stroke Registry. Notably, they report a 61% greater adjusted odds of treatment with thrombolysis among patients treated with telestroke but a 44% lower adjusted odds of achieving American Heart Association Target: Stroke–recommended treatment within 60 minutes.^5^

The analysis included 785 patients treated with telestroke and 2251 patients treated without telestroke who presented within 4 hours of last known well and had no documented contraindication to thrombolysis.^4^ Telestroke use was captured across more than 50 hospitals throughout Michigan over a 2-year period. The authors included robust assessment of covariates, accounting for variation at the patient and hospital level, and analyzed meaningful, actionable outcomes such as door-to-needle (DTN) and door-in-door-out (DIDO) times. After adjusting for patient and hospital characteristics, telestroke DTN times were 6.6 minutes longer than times for patients not treated with telestroke. Among 255 patients requiring interhospital transfer, DIDO time was 47 minutes longer for those treated with telestroke.

Notably, in this analysis, patients without telestroke activation were more commonly treated at metropolitan teaching hospitals with neurology residency programs and with higher levels of stroke center certification,^1^ facilities known for more robust resources, faster treatment times, and better patient outcomes.^6^ Thus, these results cannot directly quantify for us the benefit of telestroke associated with a setting with no access to expertise. An alternative comparison of patients with and without telestroke activations limited to those in lower-resourced settings may have led to different findings and potentially even further highlighted the benefits of telestroke.

The findings of a greater likelihood of treatment with thrombolysis but slower treatment and transfer times highlight both the strengths of telestroke and critical areas for improvement. More than 25 years after the evidentiary trials, thrombolysis remains underused.^7^ Ongoing expansion of access to thrombolysis and thrombectomy is essential. However, the benefit of stroke reperfusion therapy is highly time dependent. Hub-and-spoke telestroke networks must strive toward and be held accountable for achieving Target: Stroke–recommended DTN and DIDO times.^5^ Increased accountability at hub hospitals for the care delivery and outcomes in their networks has been proposed as a strategy for motivating investment in improved performance in local and regional stroke systems of care.^8^ Improvement efforts should address processes at both hub and spoke hospitals. For example, in academic medical centers, redundancy, such as requiring attending physicians to verify trainee assessments, can further education but delay time-sensitive clinical decision-making and processes such as transfers. Spoke hospitals across health system lines should also be empowered to collaborate and share best practices with peer institutions facing similar challenges. With continued assessment of processes and outcomes across stroke systems of care, and collaboration beyond traditional health system boundaries, we can further improve outcomes for patients treated with telestroke.

## References

[1] Wechsler LR, Demaerschalk BM, Schwamm LH, ; American Heart Association Stroke Council; Council on Epidemiology and Prevention; Council on Quality of Care and Outcomes Research. Telemedicine quality and outcomes in stroke: a scientific statement for healthcare professionals from the American Heart Association/American Stroke Association. Stroke. 2017;48(1):e3–e25. doi:10.1161/STR.0000000000000114 27811332

[2] Adeoye O, Nyström KV, Yavagal DR, Recommendations for the establishment of stroke systems of care: a 2019 update. Stroke. 2019;50(7):e187–e210. doi:10.1161/STR.0000000000000173 31104615

[3] Zachrison KS, Cash RE, Adeoye O, Estimated population access to acute stroke and telestroke centers in the US, 2019. JAMA Netw Open. 2022;5(2):e2145824. doi:10.1001/jamanetworkopen.2021.45824 35138392 PMC8829668

[4] Stamm B, Whitney RT, Royan R, Telestroke and timely treatment and outcomes in patients with acute ischemic stroke. JAMA Netw Open. 2025;8(9):e2534275. doi:10.1001/jamanetworkopen.2025.34275 41004145 PMC12475942

[5] Phase III Target: Stroke: higher goals for greater good. American Heart Association. Accessed August 20, 2025. https://www.heart.org/-/media/Files/Professional/Quality-Improvement/Target-Stroke/Target-Stroke-Phase-III/TS-Phase-III-5-6-19/FINAL5619-Target-Stroke-Phase-3-Brochure.pdf

[6] Man S, Zhao X, Uchino K, Comparison of acute ischemic stroke care and outcomes between comprehensive stroke centers and primary stroke centers in the United States. Circ Cardiovasc Qual Outcomes. 2018;11(6):e004512. doi:10.1161/CIRCOUTCOMES.117.004512 29794035 PMC5978771

[7] Sun P, Zheng L, Lin M, Persistent inequities in intravenous thrombolysis for acute ischemic stroke in the United States: results from the Nationwide Inpatient Sample. J Am Heart Assoc. 2024;13(9):e033316. doi:10.1161/JAHA.123.033316 38639371 PMC11179951

[8] Zachrison KS, Reeves MJ. Stroke systems of care 2.0: moving toward definability, accountability, and equity. Stroke. 2024;55(4):1094–1097. doi:10.1161/STROKEAHA.123.044263 38197264 PMC10978270

